# Structural insights into protein folding, stability and activity using *in vivo* perdeuteration of hen egg-white lysozyme

**DOI:** 10.1107/S2052252521001299

**Published:** 2021-03-06

**Authors:** Joao Ramos, Valerie Laux, Michael Haertlein, Elisabetta Boeri Erba, Katherine E. McAuley, V. Trevor Forsyth, Estelle Mossou, Sine Larsen, Annette E. Langkilde

**Affiliations:** a Life Sciences Group, Institut Laue–Langevin, 71 Avenue des Martyrs, 38000 Grenoble, France; b Partnership for Structural Biology (PSB), 71 Avenue des Martyrs, 38000 Grenoble, France; c Department of Drug Design and Pharmacology, University of Copenhagen, Universitetsparken 2, DK-2100 Copenhagen, Denmark; d Institut de Biologie Structurale, Université de Grenoble Alpes, CEA, CNRS, 71 Avenue des Martyrs, 38000 Grenoble, France; e Diamond Light Source, Didcot OX11 0DE, United Kingdom; f Paul Scherrer Institute, Forschungsstrasse 111, 5232 Villigen, Switzerland; g Faculty of Natural Sciences, Keele University, Newcastle-under-Lyme ST5 5BG, United Kingdom; h Department of Chemistry, University of Copenhagen, Universitetsparken 5, DK-2100 Copenhagen, Denmark

**Keywords:** perdeuterated lysozyme, HEWL, isotope effect, protein refolding, biophysical characterization, X-ray crystallography, protein structure, structural biology

## Abstract

This structural and biophysical study exploited a method of perdeuterating hen egg-white lysozyme based on the expression of insoluble protein in *Escherichia coli* followed by in-column chemical refolding. This allowed detailed comparisons with perdeuterated lysozyme produced in the yeast *Pichia pastoris*, as well as with unlabelled lysozyme. Both perdeuterated variants exhibit reduced thermal stability and enzymatic activity in comparison with hydrogenated lysozyme. The thermal stability of refolded perdeuterated lysozyme is 4.9°C lower than that of the perdeuterated variant expressed and secreted in yeast and 6.8°C lower than that of the hydrogenated *Gallus gallus* protein. However, both perdeuterated variants exhibit a comparable activity. Atomic resolution X-ray crystallographic analyses show that the differences in thermal stability and enzymatic function are correlated with refolding and deuteration effects. The hydrogen/deuterium isotope effect causes a decrease in the stability and activity of the perdeuterated analogues; this is believed to occur through a combination of changes to hydrophobicity and protein dynamics. The lower level of thermal stability of the refolded perdeuterated lysozyme is caused by the unrestrained Asn103 peptide-plane flip during the unfolded state, leading to a significant increase in disorder of the Lys97–Gly104 region following subsequent refolding. An ancillary outcome of this study has been the development of an efficient and financially viable protocol that allows stable and active perdeuterated lysozyme to be more easily available for scientific applications.

## Abstract 2

Perdeuteration and *in vitro* refolding of hen egg-white lysozyme impact protein thermal stability and activity. Deuteration appears to primarily affect enzymatic function through changes in protein dynamics, while refolding contributes to a small decrease in protein thermal stability.

## 1. Introduction   

Biomolecular deuteration is widely used in structural biology, where it plays a crucial role in techniques such as neutron macromolecular crystallography (NMX), small-angle neutron scattering (SANS), neutron reflectometry (NR), neutron spectroscopy and nuclear magnetic resonance (NMR) (Haert­lein *et al.*, 2016 ▸; Varga *et al.*, 2007 ▸). For neutron studies, the fact that deuterium (D), an isotope of hydrogen (H), possesses a positive coherent neutron scattering length and a small incoherent neutron scattering cross section, and also an integer nuclear spin, is of central importance. In NMX, perdeuteration may be used to eliminate the incoherent scattering arising from the two spin states of the H atom (incoherent scattering cross section of 80.27 barns; Sears, 1992 ▸); this allows the use of samples that are approximately one order of magnitude smaller by volume (Hazemann *et al.*, 2005 ▸) and may result in improved resolution (Blakeley, 2009 ▸). Perdeuteration enhances the visibility of the coherent signal (Bragg reflections), fully exploiting the higher coherent scattering length of deuterium (6.67 fm) in comparison with that of hydrogen (∼3.74 fm). Furthermore, perdeuteration helps to avoid cancellation effects (arising from the negative scattering length of hydrogen) that may occur for neutron Fourier maps based on data with intermediate resolution (for example *d* > 1.6 Å). In SANS, the use of deuterated samples allows contrast-matching techniques (Dunne *et al.*, 2017 ▸; Laux *et al.*, 2008 ▸; Haertlein *et al.*, 2016 ▸) to provide unique information on protein–protein (Vijayakrishnan *et al.*, 2010 ▸), protein–nucleic acid (Cuypers, Trubitsyna *et al.*, 2013 ▸) or protein–lipid (Breyton *et al.*, 2013 ▸) interactions. Sophisticated technologies have also been developed to allow the production of stealth-deuterated nanodiscs (Maric *et al.*, 2014 ▸, 2015 ▸) for the study of membrane proteins (Josts *et al.*, 2018 ▸; Nitsche *et al.*, 2018 ▸; Kehlenbeck *et al.*, 2019 ▸). In the case of NR, a wide range of research now routinely exploits the contrast enabled through the use of selective deuteration (Grage *et al.*, 2011 ▸; Moulin *et al.*, 2018 ▸; Waldie *et al.*, 2018 ▸, 2019 ▸, 2020 ▸). Deuterium labelling and reverse labelling have also been used for neutron scattering studies of the dynamics of biological macromolecules (Foglia *et al.*, 2016 ▸), in particular when coupled with hydration water dynamics (Wood *et al.*, 2013 ▸). In the case of solution-state NMR, deuterium labelling is essential for multidimensional heteronuclear NMR studies of proteins, especially high-molecular-weight proteins and macromolecular complexes. Partial deuteration simplifies the NMR spectra from the remaining ^1^H nuclei and also contributes to spectra with a higher signal-to-noise ratio owing to the effects on the relaxation of bonded or adjacent ^1^H, ^13^C and ^15^N atoms (Sattler & Fesik, 1996 ▸). While major developments in *in vivo* deuteration technologies have occurred in the last 15 years, the expression of deuterated protein is often complex and expensive and may be associated with low yields. The way in which it is carried out depends on the downstream application and on the labelling regime needed to answer the scientific questions posed (Haertlein *et al.*, 2016 ▸). In the case of neutron crystallographic applications, the goal is invariably to perdeuterate the sample so that the incoherent scattering from hydrogen is removed from the recorded data to the maximum possible extent.

Hen egg-white lysozyme (HEWL) was the first enzyme structure to be solved by X-ray crystallography (Blake *et al.*, 1965 ▸), and has subsequently become a widely used model in structural biology in a variety of contexts including protein-folding studies (Miranker *et al.*, 1991 ▸, 1993 ▸; Radford *et al.*, 1992 ▸; Wildegger & Kiefhaber, 1997 ▸) and crystallization (Durbin & Feher, 1986 ▸; McPherson & DeLucas, 2015 ▸; Darmanin *et al.*, 2016 ▸). It is a small (129 residues, 14.3 kDa) and stable protein that in its hydrogenated form can be acquired at low cost and crystallized in numerous space groups under well known conditions. HEWL is a hydrolase from *Gallus gallus* that cleaves the 1,4-β-linkages between *N*-acetylmuramic acid and *N*-acetyl-d-glucosamine residues in peptido­glycan. The recombinant production of HEWL in *Escherichia coli* is challenged by the reductive environment of the bacterial cytosol, which prevents the correct formation of its four disulfide bridges, resulting in the formation of inclusion bodies. The use of the yeast *Pichia pastoris* has been investigated as an expression system for the production of recombinant HEWL (Liu *et al.*, 2003 ▸; Li *et al.*, 2012 ▸; Mine *et al.*, 1999 ▸; Campbell *et al.*, 2018 ▸). While this approach results in the production of high-quality protein, the low yield is problem­atic for neutron crystallographic and spectroscopic applications. For this reason, we developed an approach whereby large quantities of insoluble protein were produced as inclusion bodies in *E. coli*, followed by an optimized refolding process, significantly improving the yield.

Using this strategy, large amounts of correctly folded perdeuterated HEWL (D-HEWL) can be obtained at a financially viable level. Of particular interest is the fact that the refolded perdeuterated lysozyme from *E. coli* (D-HEWL_EC_) provides important insights into the structural and biophysical properties of HEWL when compared with those of the perdeuterated analogue produced in *P. pastoris* (D-HEWL_PP_) and those of the commercially available non­recombinant hydrogenated protein (H-HEWL). These variants are identical in primary structure, with the exception of an additional glycine at the N-terminus of D-HEWL_EC_.

Atomic resolution X-ray structures have been determined for all three variants using triclinic crystals obtained in closely comparable conditions. The effect of deuteration on reduced thermal stability and activity is noted. The structural analyses highlight subtle but important differences that are related to the decrease in the thermal stability of D-HEWL_EC_; these differences are of significance for protein folding (Biter *et al.*, 2016 ▸), enzymatic activity (Lea & Simeonov, 2012 ▸), crystallization (Geders *et al.*, 2012 ▸; Reinhard *et al.*, 2013 ▸) and protein–ligand interactions (Bai *et al.*, 2019 ▸; Forneris *et al.*, 2009 ▸; Holdgate *et al.*, 2010 ▸; Ramos *et al.*, 2019 ▸). The improved yield (by a factor of more than three compared with that found using *P. pastoris*) paves the way for a wide range of studies that can exploit H/D isotopic substitution in this protein.

## 2. Methods   

### 2.1. Expression of D-HEWL_EC_   

Recombinant D-HEWL was overexpressed in *E. coli* BL21 (DE3) cells grown in a Labfors 2.3 l computer-controlled fermenter (Infors, France) using a high cell-density culture (HCDC) strategy. Transformation of chemically competent cells with the vector pET-28a(+) (GenScript) containing codon-optimized cDNA for HEWL expression (Supplementary Fig. S1) was performed by heat shock. Using a lysogeny broth (LB) solid medium supplemented with 40 µg ml^−1^ kanamycin (catalogue No. 60615; Sigma–Aldrich), transformed cells were selected. The cells containing the vector were then adapted to hydrogenated Enfors minimal medium containing hydrogenated glycerol and kanamycin. The cells were further adapted to fully deuterated Enfors medium supplemented with d_8_-glycerol (catalogue no. DLM-558-PK; Eurisotop) and antibiotic. 100 ml precultures were prepared to inoculate 1.4 l minimal medium in the fermenter. During batch and fed-batch phases, the pD (pD = pH_measured_ + 0.4; Glasoe & Long, 1960 ▸) was maintained at 6.4 by adding NaOD. The gas-flow rate of sterile-filtered air was 0.5 l min^−1^. Stirring was adjusted to ensure a dissolved oxygen level of 30%. The initial glycerol supply was consumed during the batch phase. The cells were then fed continuously with fresh feeding solution containing 12% d_8_-glycerol in an exponential manner (fed-batch phase). When the cell density reached an OD of 10, recombinant protein expression was induced by adding IPTG to a final concentration of 1 m*M*. The cells were harvested after 24 h of induction. The final volume of cell culture extracted from the fermenter was approximately 1.8 l.

### 2.2. Inclusion-body separation of D-HEWL_EC_   

The inclusion bodies were purified in a centrifugation-based approach, with several washing steps to remove nucleic acids, lipids and other contaminants. After pelleting the *E. coli* cells, lysis was promoted by sonication with a Vibra-Cell ultrasonic liquid processor (VCX-750-220, Sonics & Materials), performing three cycles of 30 s at amplitude 0.8 in a buffer consisting of 0.1 *M* Tris–HCl pH 8.45, 150 m*M* NaCl, 25 m*M* EDTA, 25 m*M* DTT, 0.5% Triton X-100. The suspension was centrifuged at 10 080*g* for 1 h at 4°C to separate the soluble and insoluble fractions. The supernatant was discarded and the pellet was solubilized in 0.1 *M* Tris–HCl pH 8.45, 150 m*M* NaCl, 25 m*M* EDTA, 25 m*M* DTT, 1 *M* guanidine–HCl, 1% Triton X-100 using a homogenizer (D1000, Benchmark). The suspension was sonicated three times for 10 s at amplitude 0.4 and was then centrifuged at 22 680*g* for 30 min at 4°C. This washing step was performed six times, with Triton X-100 excluded from the buffer in the last two cycles.

### 2.3. Purification of D-HEWL_EC_   

The inclusion bodies were solubilized in 0.1 *M* Tris–HCl pH 8.45, 150 m*M* NaCl, 25 m*M* EDTA, 25 m*M* DTT, 6 *M* guanidine–HCl using a homogenizer. The suspension was sonicated three times for 10 s at amplitude 0.4 and then centrifuged at 22 680*g* for 1 h at 4°C. The soluble fraction was collected and filtered through 0.4 µm filters. Purification of unfolded protein was performed by gel filtration on a HiLoad 16/600 Superdex 200 pg column (GE Healthcare) equilibrated in the same buffer. Protein fractions of 5 ml were diluted to avoid saturation of the UV detector of the HPLC and were injected into the column, running an isocratic flow at 1.0 ml min^−1^. Pure protein eluted at 0.6–0.7 column volumes (CV). The fractions of pure protein collected were frozen at −80°C until the refolding procedure.

### 2.4. Refolding of D-HEWL_EC_   

Denatured protein was refolded at room temperature in a size-exclusion chromatography (SEC) setup using a HiLoad 16/600 Superdex 200 pg column equilibrated with 0.1 *M* Tris–HCl pH 8.45, 2 *M* urea, 1 m*M* EDTA, 3 m*M* reduced glutathione, 0.3 m*M* oxidized glutathione as described by Batas & Chaudhuri (1996 ▸). 5 ml injections of pure unfolded HEWL at concentrations of 1–2 mg ml^−1^ were performed in each run; the isocratic flow was set to 0.1 ml min^−1^, resulting in monomeric HEWL fractions being collected at 0.9 CV.

The protein buffer was exchanged to 50 m*M* sodium acetate pD 4.5 in D_2_O by desalting using two coupled HiTrap 5 ml desalting columns (GE Healthcare). Injections of 2.5 ml of protein at 0.6 mg ml^−1^ were performed. The protein was subsequently concentrated to 20 mg ml^−1^ for crystallization experiments.

### 2.5. Expression and purification of D-HEWL_PP_   

The expression of D-HEWL_PP_ was achieved as described by Campbell *et al.* (2018 ▸). Since the protein was secreted into the extracellular medium, the supernatant was recovered upon cell pelleting. The supernatant was diluted by the addition of 50 m*M* Tris–HCl pH 7.8 buffer to achieve a solution conductivity of below 10 mS cm^−1^. Pure protein was obtained by ion-exchange chromatography (IEC) using an SP-Sepharose column (GE Healthcare) and elution with a 30 ml NaCl gradient from 0 to 1 *M* in 50 m*M* Tris–HCl pH 7.8 buffer. Following the same approach as the final buffer exchange of D-HEWL_EC_, the D-HEWL_PP_ buffer was exchanged to 50 m*M* sodium acetate pD 4.5 in D_2_O by desalting. The protein was concentrated to 30 mg ml^−1^ for crystallization experiments.

### 2.6. Mass spectrometry (MS)   

MS under denaturing conditions was utilized to assess the mass of the intact deuterated proteins and their degree of labelling. Specifically, liquid-chromatography/electrospray ionization mass spectrometry (LC/ESI-MS) on a 6210 TOF mass spectrometer coupled to an HPLC system (1100 series, Agilent Technologies) was performed. Data acquisition was carried out in positive-ion mode, and mass spectra were recorded in the 300–3200 *m*/*z* range. The following experimental settings were utilized: the ESI source temperature was set to 300°C, N_2_ was used as a drying gas (with a flow rate of 7 l min^−1^) and as a nebulizer gas (using a pressure of 69 kPa) and the capillary needle voltage was 4 kV. Voltages in the first part of the instrument were set as follows: the voltage of the fragmentor was 250 V and that of the skimmer was 60 V. The acquisition rate was one spectrum per second. Instrument pressure values were typically 2.33 Torr (rough vacuum) and 4.6 × 10^−7^ Torr (TOF vacuum). The mass spectrometer was calibrated with tuning mix (ESI-L, Agilent Technologies). The HPLC mobile phases were prepared with HPLC-grade solvents. The mobile phase *A* composition was 95% H_2_O, 5% acetonitrile (ACN), 0.03% trifluoroacetic acid (TFA). The mobile phase *B* composition was 95% ACN, 5% H_2_O, 0.03% TFA.

As partial D-to-H back-exchange would be possible during the experiment, both samples were dialyzed against 50 m*M* sodium acetate pH 4.5 buffer in H_2_O prior to the MS experiment to ensure full back-exchange and thus allow the evaluation of the number of D atoms in all non-exchangeable positions.

Just before the analysis, the samples were diluted in 0.03% TFA to obtain a concentration of 5 µ*M* and a volume of 20 µl. The samples were loaded into glass vials, which were placed on a sample loader refrigerated at 10°C. 4 µl of each sample (*i.e.* ∼20 pmol of protein) was injected into the HPLC system directly connected to the mass spectrometer. The injected sample was first trapped and desalted on an RP-C8 cartridge for 3 min at a flow rate of 50 µl min^−1^ using 100% mobile phase *A*. Afterwards, the proteins were separated on an RP-C8 column using a linear gradient from 5 to 95% mobile phase *B* for 15 min and subjected to ESI prior to the TOF detection of their *m*/*z* signals. The software *MassHunter BioConfirm* (version B.07.00; Agilent Technologies) was used to calculate masses from *m*/*z* values obtained during the MS experiments.

### 2.7. Differential scanning fluorimetry (DSF)   

DSF measurements were performed using a Prometheus instrument (NanoTemper). The setup included a temperature ramp from 20 to 95°C with increments of 1.0°C min^−1^, following unfolding by the intrinsic fluorescent signal from the tryptophan residues (six tryptophans in HEWL). Lyophilized H-HEWL powder was dissolved in 50 m*M* sodium acetate pD 4.5 in D_2_O to match the conditions of D-HEWL_EC_ and D-HEWL_PP_. The experiment was repeated in the hydrogenated buffer of the activity assay, where the samples were diluted in 0.1 *M* sodium phosphate pH 7.5, 0.1 *M* NaCl, 2 m*M* NaN_3_ in H_2_O in a ratio of at least 1:50. The results presented correspond to samples at concentrations of 0.3 mg ml^−1^ with a 40% excitation power and were obtained for at least two HEWL preparations as duplicate or triplicate measurements for every condition.

### 2.8. HEWL activity assays   

The activity assays were performed based on the work of Shugar (1952 ▸). The activity is followed by the absorbance at 450 nm at 25°C, with measurements every minute for 20 min. Nunc 96-well flat-bottom plates (Thermo Fisher Scientific) were used with each sample in triplicate, including negative controls without protein. The 100 µl samples used for these experiments comprised 50 µl protein sample at 0.2 mg ml^−1^ and 50 µl *Micrococcus lysodeikticus* cell suspension in H_2_O with 0.1 *M* sodium phosphate pH 7.5, 0.1 *M* NaCl, 2 m*M* NaN_3_. After averaging triplicates of each experiment, the activity curves were plotted against time, and the linear phase (*R* ^2^ > 0.91) corresponding to the first 8 min of reaction was considered to retrieve the initial velocities. Standard deviations were derived from three separate experiments and a *t*-test was performed for each pair of results to assess the significance of the homoscedastic hypothesis, meaning the probability of the pairs of measured values being equal.

### 2.9. Protein crystallization   

H-HEWL (catalogue No. L6876; Sigma–Aldrich) was crystallized in the triclinic form in batch-like conditions using a precipitation step as described by Vidal *et al.* (1999 ▸). 5 µl drops were prepared consisting of 2.5 µl H-HEWL at 20 mg ml^−1^ dissolved in deionized water and 2.5 µl 0.4 *M* NaNO_3_, 50 m*M* sodium acetate pH 4.5. Under these conditions, monoclinic crystals readily formed at room temperature. To obtain the triclinic crystal form, the crystallization plate was stored at 4°C overnight and then subsequently kept at 18°C. During the cold storage, crystals of both the triclinic and monoclinic forms nucleate. When the temperature is raised, the less stable monoclinic form dissolves, leaving almost exclusively nuclei of the triclinic form (Legrand *et al.*, 2002 ▸). Triclinic crystals appeared after three days.

Triclinic crystals of D-HEWL were obtained by initial microseeding using triclinic H-HEWL seeds from a crystal in 100% D_2_O buffer. The seed solution was made by crushing the crystal in 0.3 *M* NaNO_3_, 50 m*M* sodium acetate pD 4.5. Subsequently, the solution was transferred to an Eppendorf tube containing a zirconium silicate ceramic seed bead (Hampton Research) and vortexed to produce microseeds. Seed stocks of 1:100 and 1:1000 dilutions were used in the crystallization experiments. Sitting drops of 5.5 µl were prepared by microbatch under oil and stored at 18°C. The drops consisted of 2.5 µl D-HEWL_EC_ at 20 mg ml^−1^ or D-HEWL_PP_ at 30 mg ml^−1^, 2.5 µl 0.3 *M* NaNO_3_, 50 m*M* sodium acetate pD 4.5 and 0.5 µl of the H-HEWL seed solution. Triclinic crystals of D-HEWL of up to 0.1 mm^3^ were obtained within one week.

### 2.10. X-ray data collection, processing and model refinement   

Synchrotron X-ray diffraction data were collected at 100 K from crystals of H-HEWL, D-HEWL_EC_ and D-HEWL_PP_. The data collections were performed on beamline I03 at Diamond Light Source (DLS), UK and on BioMAX at MAX IV, Sweden (Table 1 ▸). Crystals of approximately 0.1 mm^3^ were cooled in cryoprotectant solutions of 25–35%(*v*/*v*) glycerol or d_8_-glycerol with 0.3 *M* NaNO_3_ and 50 m*M* sodium acetate pH/pD 4.5 in H_2_O for H-HEWL or D_2_O for both D-HEWL forms. Due to the low triclinic crystal symmetry, the data sets were measured in two different κ orientations to improve the completeness of the data. The data were reduced, merged and scaled using *XDS* (Kabsch, 2010 ▸). Initial phases were estimated by molecular replacement in *Phenix* (Liebschner *et al.*, 2019 ▸), using the structure deposited in the Protein Data Bank (PDB; Berman *et al.*, 2000 ▸) as entry 4yeo (Shabalin *et al.*, 2015 ▸), stripped of ligands and water molecules, as a starting model. Model refinement was performed using *Phenix* (Liebschner *et al.*, 2019 ▸), with the same set of reflections flagged for the *R*_free_ calculation. Model building was achieved using *Coot* (Emsley *et al.*, 2010 ▸). The D-HEWL_EC_ model from a late stage of refinement was used for the initial refinement of D-HEWL_PP_ and H-HEWL to maintain the labelling of residue disorder as well as of water molecules and ions. H/D atoms were added to the models as riding atoms in ideal positions. The occupancy of water molecules and ions was refined for atoms with *B* factors above 20 Å^2^ and was otherwise fixed to 1. Water molecules which displayed a density lower than 1.5 σ in the 2*F*_o_ − *F*_c_ electron-density map were removed from the models.

### 2.11. X-ray structure analysis and comparison   

Structural alignment of the entire protein chains was achieved with the *CEALIGN* plugin (Shindyalov & Bourne, 1998 ▸) using the C^α^ atoms from 128 residues, while alignment of the Lys97–Gly104 region (using all atoms) was performed with the *SUPER* function of *PyMOL* (version 2.0; Schrödinger). *EDSTATS* (Tickle, 2012 ▸) from the *CCP*4 suite (Winn *et al.*, 2011 ▸) was employed to evaluate the quality of the models according to the data, allowing the identification of residues that may not have been reliably modelled for further analysis. The combination of cutoffs considered was 90% for the RSCC, 1σ for the sample RSZO and −3σ and +3σ for RSZO− and RSZO+, respectively. Hydrogen-bond analysis was performed using *HBPLUS* (McDonald & Thornton, 1994 ▸). Results that included intra-residue interactions and residues that were not reliably modelled according to the metrics from *EDSTATS* (Tickle, 2012 ▸) were not considered for the comparison between models, with the exception of Thr89 from D-HEWL_PP_, which participates in an extensive hydrogen-bond network involving His15, Asp87 and Asn93. The graphical representations presented here were made in *PyMOL*.

## 3. Results   

### 3.1. Increased yield by refolding from inclusion bodies   

**Figure 1:**
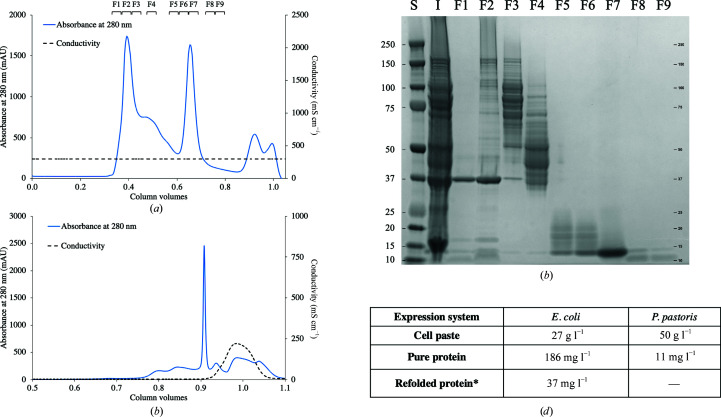
The expression of insoluble D-HEWL in *E. coli* followed by refolding increases the yield of protein production by more than threefold. (*a*) Chromatogram from the denaturing SEC, yielding pure unfolded D-HEWL_EC_, which eluted at 0.6–0.7 CV. (*b*) Fractions from the denaturing SEC on a 12% SDS–PAGE gel. Lane S, Precision Plus Protein Dual Xtra Standards (Bio-Rad); lane I, injected sample of unpurified D-HEWL_EC_ from the inclusion-body washing steps; lanes F1–F9, collected fractions from the denaturing SEC as indicated at the top of (*a*). Fractions F5–F7 were used in subsequent refolding experiments. (*c*) Refolding SEC chromatogram, where monomeric D-HEWL elutes at 0.9 CV. The fractions eluting before and after the monomeric refolded D-HEWL_EC_ are likely to be misfolded or oligomeric and partially unfolded forms of D-HEWL_EC_, respectively. This is followed by the elution of the guanidine–HCl and the DTT from the denaturing buffer, as shown by the increase in conductivity. (*d*) Comparison of the D-HEWL expression yields between the two systems, *E. coli* and *P. pastoris*. *Considering an average refolding yield of 20%, the final yield of D-­HEWL_EC_ production is 37 mg l^−1^ without further denaturing and refolding of the misfolded, oligomeric and partially unfolded fractions.

Inclusion bodies from D-HEWL_EC_ expression were separated from insoluble contaminants, as shown by SDS–PAGE of the supernatants from the washing steps (Supplementary Fig. S2). The untagged D-HEWL_EC_ was further purified by gel filtration, eluting as a single peak around 0.6–0.7 CV, with fractions F5–F7 being collected for the refolding step [Figs. 1 ▸(*a*) and 1 ▸(*b*)].

D-HEWL_EC_ was refolded in-column using a low flow rate of 0.1 ml min^−1^, which allowed desalting of the unfolded protein and separation of the monomeric and oligomeric, misfolded and partially unfolded fractions [Fig. 1 ▸(*c*)]. The fractions of refolded D-HEWL in refolding buffer and in deuterated protein buffer are shown on a 12% SDS–PAGE gel in Supplementary Fig. S3. The refolding yields were impacted by the fact that the molecular weight of HEWL is close to the lower exclusion limit of the gel-filtration column (*M*_r_ = 10 kDa), which hindered optimal separation of the monomeric protein fraction from the denaturing buffer. Injections of 6.5 mg unfolded protein resulted in average refolding yields of 20%. The expression, purification and refolding strategy yielded 186 mg of pure protein per litre of culture on average, from which, considering a consistent refolding yield of 20%, 37 mg was recovered in a native-like state. These results represent more than a threefold increase in D-HEWL production compared with the *P. pastoris* system [Fig. 1 ▸(*d*)].

### 3.2. All non-exchangeable H positions are fully deuterated in both D-HEWL_EC_ and D-HEWL_PP_   

The deuteration level of D-HEWL_EC_ and D-HEWL_PP_ was assessed by LC/ESI-MS. Prior to the MS experiments, both samples were dialyzed against 50 m*M* sodium acetate pH 4.5 buffer in H_2_O to avoid partial back-exchange during the experiment. Therefore, the expected masses included D in all non-exchangeable positions (*i.e.* bound to C) and, with full back-exchange, H in all labile positions (Table 2 ▸).

D-HEWL_EC_ has 130 residues, with one additional glycine at the N-terminus compared with the other HEWL variants studied (Supplementary Fig. S1), resulting in differences in the expected masses. The masses observed by MS of 15 060 and 15 005 Da (Supplementary Fig. S4) for D-HEWL_EC_ and D-HEWL_PP_, respectively, closely match the expected values (Table 2 ▸) and verify the successful replacement of H atoms by D atoms in non-exchangeable positions. D-HEWL_EC_ shows a minor difference of 4 Da between the expected and the observed masses, which shows that 99.4% of all non-exchangeable positions are occupied by D. The D-HEWL_PP_ observed mass exactly matched the expected value of the fully deuterated form.

### 3.3. Perdeuterated variants of lysozyme are stable and active   

**Figure 2:**
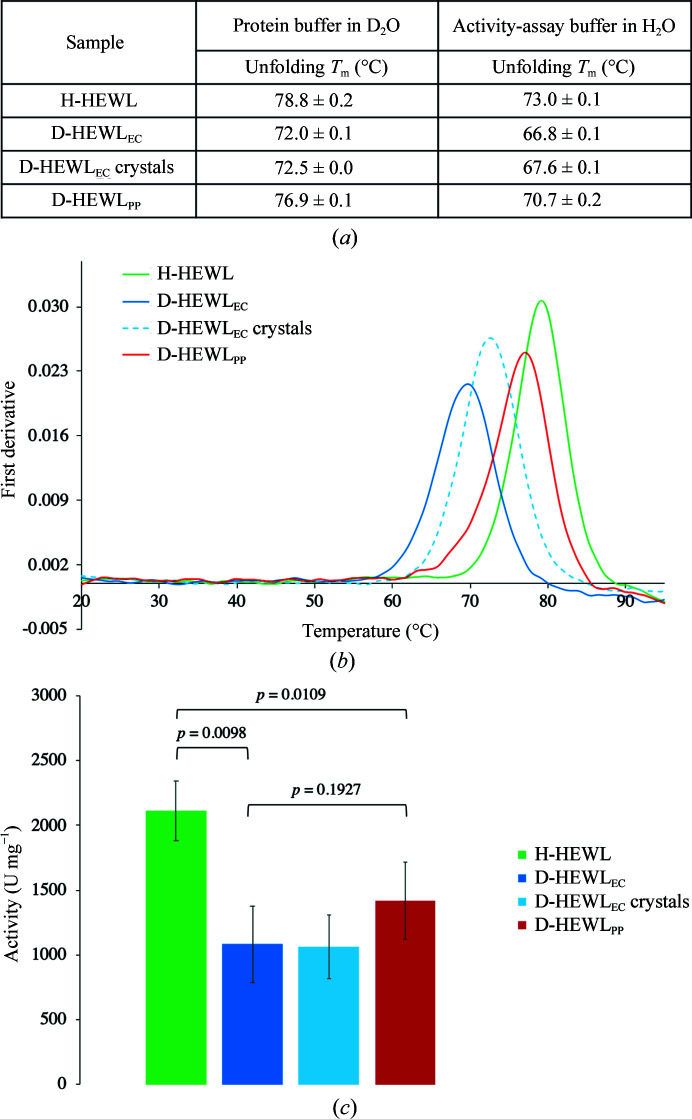
Stability and activity of the HEWL variants. (*a*) *T*_m_ values for H-HEWL, D-HEWL_EC_ (before and after crystallization) and D-HEWL_PP_ in deuterated protein buffer (50 m*M* sodium acetate pD 4.5) and in hydrogenated activity-assay buffer (0.1 *M* sodium phosphate pH 7.5, 0.1 *M* sodium chloride, 2 m*M* sodium azide). (*b*) Thermal unfolding curves (first derivative against temperature) of H-HEWL (green), D-­HEWL_EC_ before crystallization (continuous dark blue line) and after crystallization (dashed light blue line) and D-HEWL_PP_ (red) in deuterated protein buffer. (*c*) Enzymatic activity of H-HEWL (green), D-HEWL_EC_ (dark blue, before crystallization; light blue, after crystallization) and D-HEWL_PP_ (red) in the hydrogenated activity-assay buffer. The *p*-values represent the significance of the homoscedastic hypothesis, meaning the probability of the pairs of measured values being equal.

DSF assays were performed to retrieve information on the folding and stability of D-HEWL_EC_ and D-HEWL_PP_ using H-HEWL as a reference. Results were obtained using the same deuterated buffer (50 m*M* sodium acetate pD 4.5 in D_2_O) and showed that both variants of D-HEWL are thermally less stable than H-HEWL [Figs. 2 ▸(*a*) and 2 ▸(*b*)]. The refolded D-HEWL_EC_ is less thermally stable than D-HEWL_PP_, with a difference in melting temperature of 4.9°C. Moreover, compared with H-HEWL, the refolded D-HEWL_EC_ shows a decrease in thermal stability of 6.8°C. If the D-HEWL_EC_ was not completely separated from denaturing salts upon refolding, a small population of misfolded protein could potentially be present in the sample. To test this, D-HEWL_EC_ crystals were washed and dissolved in protein buffer (from now on referred to as D-HEWL_EC_ after crystallization) and analyzed by DSF [Figs. 2 ▸(*a*) and 2 ▸(*b*)]. With differences of less than 1°C observed between D-HEWL_EC_ before and after crystallization, it was concluded that the lower thermal stabil­ity was not attributable to the presence of misfolded protein in the D-HEWL_EC_ sample.

The enzymatic activities of the D-HEWL variants were also assessed. As part of this, DSF measurements were performed in activity-assay buffer (0.1 *M* sodium phosphate buffer pH 7.5 in H_2_O with 0.1 *M* NaCl and 2 m*M* NaN_3_). A systematic decrease in stability of all of the samples was observed in this buffer [Fig. 2 ▸(*a*)]. The D-HEWL variants are less active than H-HEWL (D-HEWL_EC_ and D-HEWL_PP_ exhibited 51% and 67% of the activity of H-HEWL, respectively; both were significantly different, with *t*-test *p* values of <0.05) [Fig. 2 ▸(*c*)]. Conversely, the activity difference between D-HEWL_EC_ and D-HEWL_PP_ is not significant (*p* = 0.19). As for the thermal stability, no significant differences were observed between D-HEWL_EC_ before and after crystallization.

### 3.4. Structural similarities and differences   

Atomic resolution X-ray diffraction data were collected for all three variants: H-HEWL, D-HEWL_EC_ and D-HEWL_PP_. The data sets all extended to at least 1.00 Å resolution, although, as evident from the merging statistics (Table 1 ▸), the resolution cutoff was limited by the experimental geometry (detector distance and coverage) and not by the diffraction power of the crystals. Given the low symmetry of the *P*1 space group, a data-collection strategy with sweeps collected in two distinct crystal orientations (different κ angles) was implemented. An overall completeness of greater than 90% was obtained to a resolution of 1.00 Å. Refinement of the three variants of lysozyme provided the basis for comparison of the features and differences between the structures. The quality and resolution of the diffraction data allowed the visualization of elusive structural detail, including side-chain and main-chain disorder, and the interpretation of complex hydrogen-bonding patterns and their underlying structural dynamics.

#### 3.4.1. Secondary and tertiary structures are retained   

Numerous structures of HEWL are available in the PDB, representing a multitude of crystallization conditions, different space groups, ligands, humidity levels, mutations *etc.*, but a benchmark in this large pool of structures is the *P*1 structure refined to 0.65 Å reesolution by Wang *et al.* (2007 ▸) (PDB entry 2vb1). With a r.m.s.d. of 0.23 Å between the C^α^ atoms, the three-dimensional structure of H-HEWL obtained in our study closely matches this model. There are some differences between the disorder modelled in the two structures, which may reflect the difference in resolution of the corresponding data sets.

In order to perform a comparison of the structure of H-HEWL with the structures of the two D-HEWL variants, the model of H-HEWL was obtained from similar crystallization conditions, data-collection and refinement parameters and resolution limits to those for the D-HEWL structures.

**Figure 3:**
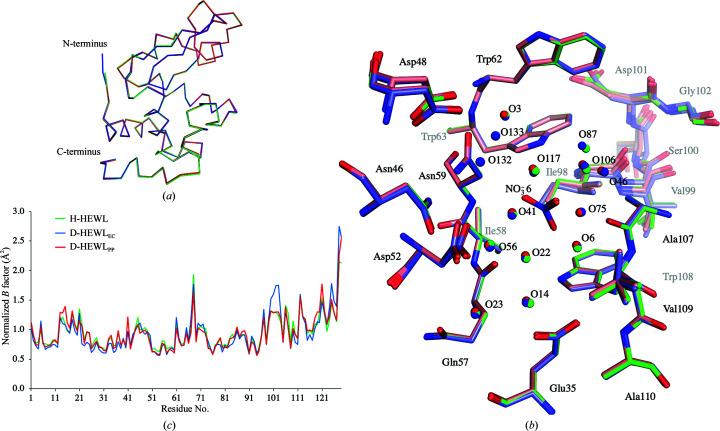
The overall structures and normalized *B* factors of the three HEWL variants. (*a*) Ribbon representation of the structurally aligned models of H-HEWL (green), D-HEWL_EC_ (blue) and D-HEWL_PP_ (red). (*b*) The active site and the polysaccharide-binding cleft shown for all three molecules: H-HEWL (green), D-HEWL_EC_ (blue) and D-HEWL_PP_ (red). (*c*) Plot of the normalized residue-averaged *B* factors from the H-HEWL (green), D-HEWL_EC_ (blue) and D-HEWL_PP_ (red) models.

The structural alignment based on C^α ^ atoms between the three HEWL variants showed a high degree of similarity, with an r.m.s.d. of 0.11 Å for both D-HEWL_EC_ and D-HEWL_PP_ in comparison with H-HEWL [Fig. 3 ▸(*a*)]. The conserved tertiary and secondary structures indicate that perdeuteration did not have a significant impact on the overall protein fold, as has been demonstrated in many neutron crystallographic studies of other proteins (Artero *et al.*, 2005 ▸; Haupt *et al.*, 2014 ▸; Langan *et al.*, 2014 ▸; Cuypers, Mason *et al.*, 2013 ▸; Yee *et al.*, 2019 ▸; Liu *et al.*, 2007 ▸; Koruza *et al.*, 2019 ▸). The r.m.s.d. between the two D-HEWL variants was 0.13 Å, suggesting that the refolding process had little effect on the global protein fold.

#### 3.4.2. Alternate conformations and hydrogen-bond patterns   

The atomic resolution X-ray data enabled a detailed description of backbone and side-chain disorder. Alternate conformations were modelled for approximately 30% of the protein residues. Overall, the structures exhibited similar disorder patterns; the only exceptions were residues Glu7, Asn19, Ser24, Gln41, Thr89 and Gln121 (Supplementary Fig. S5). Given the high resolution of the X-ray data, the structural analysis includes a comparison of hydrogen bonds in the three structures, as this is of central interest for an understanding of differences in thermal stability.

Even at this high resolution, the electron-density maps in specific regions do not allow unambiguous modelling. Thus, the results from *HBPLUS* (McDonald & Thornton, 1994 ▸) were filtered considering the RSCC and RSZO metrics from *EDSTATS* (Tickle, 2012 ▸; details are shown in Supplementary Figs. S6–S8) to ensure the reliability of the subsequent analysis. The following residues did not comply with the applied cutoffs in one or more of the structures: Gly0, Ala9, Gly26, Asn27, Ala32, Phe38, Tyr53, Leu56, Ile58, Trp62, Cys64, Thr89, Ser91, Val92, Asp101, Gly102, Asn103, Gly104, Met105, Asn106, Ala107, Cys127 and Leu129. Discrepancies in hydrogen-bond lengths larger than 0.1 Å between all three structures were considered and inspected individually.

**Figure 4:**
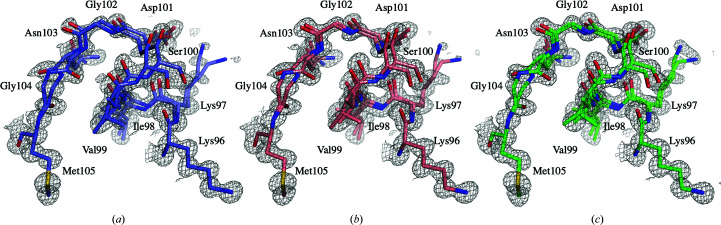
Increased disorder in the Lys97–Gly104 region of D-HEWL_EC_ compared with both D-HEWL_PP_ and H-HEWL. Representation of the backbone disorder resulting from the strain induced by the Asn103 partial peptide flip in D-HEWL_EC_ (*a*), D-HEWL_PP_ (*b*) and H-HEWL (*c*). The 2*F*_o_ − *F*_c_ electron-density maps represented are contoured at 1σ.

A comparison of the active site, with the catalytic residues Asp35 and Glu52, and the polysaccharide-binding cleft (Phillips, 1967 ▸) initially showed only minor differences in residue positions and conformations [Fig. 3 ▸(*b*)]. However, the residues Lys97–Gly104 display a high level of disorder, reflected by increased *B* values, most noticeably in the structure of D-HEWL_EC_ [Fig. 3 ▸(*c*)]. As also reported by Wang *et al.* (2007 ▸), this region contains main-chain disorder due to a partial peptide-plane flip of Asn103, which causes strain on the backbone of residues Lys97–Gly104 (Fig. 4 ▸), propagating through hydrogen-bond interactions. The occupancy of the loop conformation associated with the flipped Asn103 (conformation *B*) was 46% in D-HEWL_EC_, 38% in D-HEWL_PP_ and 33% in H-HEWL. Structural alignment of this region (Lys97–Gly104, using all atoms) showed that in comparison with H-HEWL, D-HEWL_EC_ and D-HEWL_PP_ deviate by 0.27 and 0.16 Å, respectively. Meanwhile, the r.m.s.d. between D-HEWL_EC_ and D-HEWL_PP_ was 0.21 Å.

**Figure 5:**
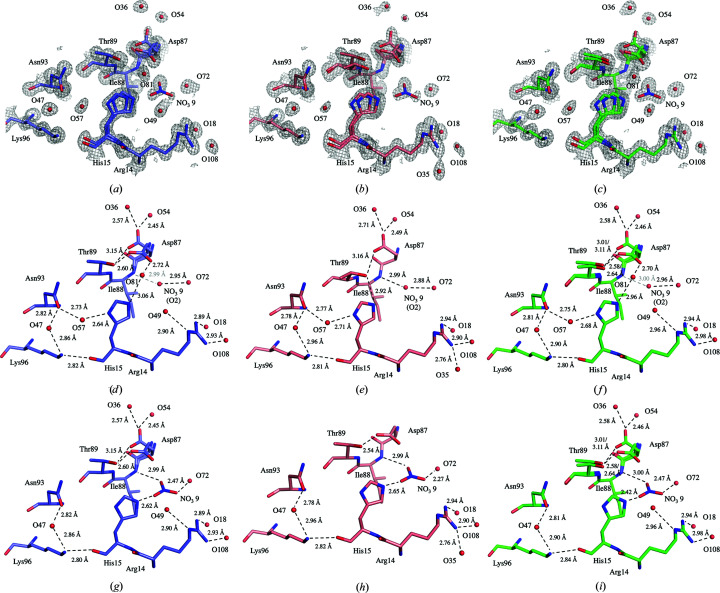
Disorder and hydrogen-bond patterns surrounding the His15 side chain. A representation is shown of the overall environment around His15 in D-­HEWL_EC_ (*a*), D-HEWL_PP_ (*b*) and H-HEWL (*c*). The 2*F*_o_ − *F*_c_ electron-density maps represented are contoured at 1σ. Highlighted hydrogen-bond interactions correlated with His15 side-chain disorder are shown for conformation *A* of D-HEWL_EC_ (*d*), D-HEWL_PP_ (*e*) and H-HEWL (*f*) and for conformation *B* of D-HEWL_EC_ (*g*), D-HEWL_PP_ (*h*) and H-HEWL (*i*).

Main-chain disorder was also observed in the Lys13–Gly16 region, which is part of the first α-helix, with variations in the Gly16 N–Lys13 O hydrogen bond (Supplementary Fig. S9). However, this relates to the disorder of the His15 side chain, together with the interaction of Lys13 with the C-terminal residue Leu129 and of Gly16 with the disordered Arg114 via crystal contacts. The alternate conformations of His15, *A* and *B*, appear to be stabilized by water-mediated hydrogen bonds to Asn93 and by a hydrogen-bond to nitrate ion 9, respectively (Fig. 5 ▸).

The most evident differences between the structures in this region are the disorder of Thr89 in H-HEWL, and more profoundly in D-HEWL_PP_, and the absence of water 81 in D-HEWL_PP_. For His15*A*, water 81 seems to be important in restraining Thr89 in H-HEWL and D-HEWL_EC_, contrary to the observation in D-HEWL_PP_. In the absence of water 81 in D-HEWL_PP_, a significant displacement of Thr89 occurs, stabilizing the His15 side chain. Furthermore, a steric clash with Thr89 appears to force flipping of the Asp87 side chain. The visualization of this extended hydrogen-bond network is supported by the similar refined occupancies of His15*A*, water 57, Thr89*B* and Asp87*B* of 47%, 34%, 39% and 41%, respectively. Meanwhile, in the H-HEWL and D-HEWL_EC_ structures, His15*A* interacts with Asn93 and Asp87 through hydrogen bonds mediated by waters 57 and 81, as shown by their refined occupancies (51% for His15*A*, 65% for water 57 and 55% for water 81 in H-HEWL; 58% for His15*A*, 70% for water 57 and 55% for water 81 in D-HEWL_EC_).

On the other hand, His15*B* in all three HEWL structures forms a hydrogen bond to nitrate ion 9, which is further stabilized by hydrogen bonds to Ile88 N and water 72. This interaction network is supported by the refined occupancies of His15*B* and nitrate ion 9 (42% and 49% in H-HEWL, 49% and 56% in D-HEWL_EC_ and 66% and 47% in D-HEWL_PP_, respectively). The low *B* factor refined for the O2 atom of this nitrate ion revealed the presence of a water molecule when the nitrate is not occupying the space (the occupancy of nitrate 9 O2 is 1, while the nitrate occupancy is refined based on N, O1 and O3). Additionally, in D-HEWL_PP_ the His15*B* side chain forms a hydrogen bond to the nitrate ion, which replaces its interaction with Thr89 and promotes the interaction of Thr89*A* with Asp87*A*, as shown by their matching occupancies of 61% and 59%, respectively.

**Figure 6:**
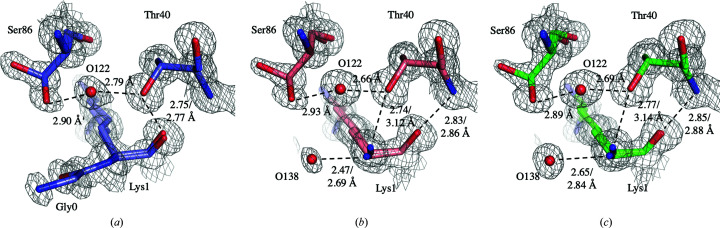
Representation of the differences in the hydrogen-bond patterns involving Lys1 and Thr40 in D-HEWL_EC_ with the additional Gly0 residue (*a*), D-­HEWL_PP_ (*b*) and H-HEWL (*c*). The 2*F*_o_ − *F*_c_ electron-density maps represented are contoured at 1σ.

The presence of Gly0 at the N-terminus of D-HEWL_EC_ influences the hydrogen-bond pattern in this region. Specifically, Gly0 cancels the Lys1 N–Thr40 OG1 interaction, instead favouring a Thr40 OG1–Lys1 O hydrogen bond (Fig. 6 ▸). Additionally, Gly0 does not interact with other protein residues and increases the disorder of the N-terminus of D-HEWL_EC_. In H-HEWL and D-HEWL_PP_, water molecule 138 occupies the position of Gly0 and enables water-mediated hydrogen bonds between Lys1 N and Ser86*B* OG.

In addition, several minor differences between the three structures were noted, where D-HEWL_EC_ in particular stands out. In D-HEWL_EC_ Asn19 was observed in a single conformation, allowing a stable Gly22 N–Asn19 O hydrogen bond of 2.96 Å, while in D-HEWL_PP_ and H-HEWL disorder was observed, with the major conformation (occupancies of 60% and 69%, respectively) resulting in a significantly longer Gly22 N–Asn19 O hydrogen bond (Supplementary Fig. S10). This variation is correlated with the alternate conformations of the Asn19 side chain in H-HEWL and D-HEWL_PP_, where the minor conformation of Asn19 participates in crystal contacts with Ser81 O, while the major conformation is involved in crystal contacts with the disordered Gln41 OE1. In D-HEWL_EC_ only the latter conformation is present, as Gln41 is ordered, resulting in a single conformation of Asn19 with the shorter intramolecular Gly22 N–Asn19 O hydrogen bond. This shorter interaction suggests a more stable 3_10_-helix between Tyr20 and Gly22 in D-HEWL_EC_, although this may be a consequence of the stable crystal contact between the side chains of Asn19 and Gln41, thus not influencing stability in solution.

In all three structures Ser81 adopts two distinct conformations, giving rise to different Leu84 N–Ser81 O hydrogen-bond lengths (Supplementary Fig. S11), where the major conformation corresponds to the shorter of the two inter­actions. However, the lower occupancy of this major conformation in D-HEWL_EC_ (66% compared with 82% in both D-HEWL_PP_ and H-HEWL), together with the larger difference in the hydrogen-bond lengths of the two conformations, indicates that the Leu84 N–Ser81 O interaction is potentially weaker in D-HEWL_EC_, destabilizing its 3_10_-helix.

Furthermore, in another 3_10_-helix (Val120–Arg125), minor variations were observed in the Arg125 NH2–Asp119 OD2 and Arg125 NH2–Gln121*B* OE1 hydrogen bonds, with the shorter Arg125 NH2–Asp119 OD2 interactions found in H-HEWL (Supplementary Fig. S12). Additionally, in D-HEWL_EC_ Arg5 forms longer side chain–main chain hydrogen bonds to Trp123 O and Arg125 O, respectively, representing a minor destabilization of the tertiary structure in comparison to D-HEWL_PP_ and H-HEWL.

## 4. Discussion   

By using an *E. coli* expression system in parallel with in-column protein refolding, it is possible to obtain a more than threefold gain in the production of D-HEWL in comparison with yields for the *P. pastoris* system. The increase in yield is proportional to the financial cost reduction of protein production, since the approximate cost per litre of *E. coli* and *P. pastoris* cultures is similar. The cost is dominated by the deuterated materials, which for D-HEWL production using the *E. coli* system is roughly 140 euros per milligram of protein, in comparison to approximately 450 euros per milligram using *P. pastoris*. Although non-optimal in separating monomeric lysozyme from denaturing salts, the SEC column used in refolding provided the highest yields when compared with analytical columns. This observation is related to difficulties in removing such high concentrations of salt and the need to separate oligomeric from monomeric fractions while injecting milligram amounts of sample. Furthermore, the yield of the protocol can be further increased by dialyzing the oligomeric, misfolded and partially unfolded fractions of D-HEWL_EC_ from refolding against denaturing buffer and reinjecting them into a refolding SEC. Complete perdeuteration of non-exchangeable sites in both D-HEWL variants was demonstrated by mass spectrometry. A similar refolding approach has been applied for the production of a perdeuterated antifreeze protein (Petit-Haertlein *et al.*, 2009 ▸), with the difference that refolding was carried out in a deuterated buffer. The refolding of perdeuterated lysozyme reported here is, to our knowledge, the first example of a perdeuterated protein exceeding 7 kDa and with multiple disulfide bonds. Refolding in D_2_O was also attempted; however, it led to a decrease in the refolding yield (data not shown) owing to reduced separation of the monomeric protein fraction and denaturing salts. This observation is likely to be due to the slower dynamics in heavy water, resulting in a delay in the elution of the folded monomeric lysozyme fraction. Additionally, using D_2_O would not be cost-effective, given the numerous refolding SEC runs that are required to obtain several milligrams of refolded protein. As the refolding of D-HEWL_EC_ was performed in H_2_O buffer, it may result in the caging of H atoms in exchangeable positions, *i.e.* exchanged during the unfolded state and then trapped upon refolding. The protein fold may keep specific regions protected from any interaction with solvent molecules; hence, to exchange these H atoms to D atoms the protein must be at least partially unfolded in D_2_O buffer. To unambiguously identify the positions occupied by caged H atoms in the protein structure, neutron crystallography or NMR experiments are required. An indication of relevant positions is found in a reverse setup, where 20 H atoms were exchanged to D using unfolding and refolding processes of H-HEWL in D_2_O (Kita & Morimoto, 2016 ▸), as observed in the neutron structure deposited in the PDB (PDB entry 6k8g; Kita & Morimoto, 2020 ▸).

Biophysical characterization of both D-HEWL variants and commercially available unlabelled HEWL shows that both D-HEWL molecules are stable and active. The perdeuterated variants showed lower thermal stability relative to the hydrogenated protein both in D_2_O and H_2_O buffers, in line with what has been reported in several biophysical studies on protein deuteration (Berns, 1963 ▸; Hattori *et al.*, 1965 ▸; Brockwell *et al.*, 2001 ▸; Meilleur *et al.*, 2004 ▸; Koruza *et al.*, 2018 ▸; Nichols *et al.*, 2020 ▸). Additionally, it seems that both hydrogenated and perdeuterated forms of HEWL have an increased transition temperature in D_2_O compared with H_2_O, as described in previous studies (Makhatadze *et al.*, 1995 ▸; Efimova *et al.*, 2007 ▸). However, the data presented here are not sufficient to draw definitive conclusions on this solvent-isotope effect, since the D_2_O and H_2_O buffers used have significantly different compositions (aimed at crystallization and activity measurements, respectively). Additionally, the presence of residual H atoms in H-HEWL, due to the limited time for H/D exchange and the limited solvent accessibility of specific protein regions to the D_2_O solvent, cannot be ruled out. The differences in protein thermal stability can be correlated with the enzymatic activities. Perdeuteration of the protein was expected to affect its dynamics and consequently its stability and activity, and in this study a decrease in stability as well as in relative activity compared with H-HEWL is observed. The differences between D-HEWL_EC_ and D-HEWL_PP_ are likely to be a consequence of the refolding procedure. The additional N-terminal glycine residue in D-HEWL_EC_ may also cause a slight destabilization of the protein. However, the activity results do not allow a conclusive correlation of the effect of refolding on activity, since under the conditions used the difference in activity between the two perdeuterated variants is not statistically significant. This further emphasizes the similarity between the D-HEWL variants and validates the refolding approach to obtain stable and active D-HEWL.

While a large number of HEWL crystal structures have been published, the detailed comparisons needed for this study of perdeuterated and hydrogenated HEWL required the growth of crystals under closely comparable conditions, with only minor variations relating to the seeding procedure and precipitant concentrations. The atomic resolution X-ray data for both perdeuterated samples, as well as for the reference unlabelled sample, have been analyzed in detail, revealing structural features that can be related to the observations on stability and activity. The crystal packing and overall structures were, as expected, found to be essentially identical, with negligible differences in the unit-cell parameters. Moreover, the nitrate and acetate ions that are essential to crystallization were located and refined in identical positions in the three models, with similar *B* factors (Table 1 ▸). However, despite the close similarity between the three structures, there are some clear variations in hydrogen-bond distances, which appear to be related to the differences in protein stability.

An important factor contributing to the reduced thermal stability of the D-HEWL structures is the effect of H/D substitution on hydrophobic interactions. As described by Hattori *et al.* (1965 ▸), deuterium-substituted nonpolar amino-acid side chains have a reduced steric requirement due to the smaller amplitudes of vibration of the C—D bond compared with C—H, leading to weaker hydrophobic interactions between the residue side chains; this has also been noted in mass-spectrometric studies (Yee *et al.*, 2016 ▸). Additionally, D_2_O has a stronger hydrophobic effect than H_2_O, leading to changes in solvation, more compact structures and a decrease in protein flexibility (Svergun *et al.*, 1998 ▸; Sasisanker *et al.*, 2004 ▸; Efimova *et al.*, 2007 ▸; Jasnin *et al.*, 2008 ▸). This is observed in the crystal structures, where a larger number of structural water molecules were identified in H-HEWL compared with both D-HEWL variants. Moreover, the molecular surface and solvent-accessible surface areas of D-HEWL_PP_ were 15 555 and 8 200 Å^2^, respectively, whereas those for H-HEWL were 15 725 and 8 274 Å^2^. The corresponding values for D-HEWL_EC_ are not directly comparable due to the presence of the additional Gly0 residue. Finally, protein dynamics are expected to be influenced by deuteration since D is twice as heavy as H, which in the case of HEWL corresponds to a mass increase of at least ∼700 Da. All of these factors play a role in the interaction with substrate molecules, since the enzymatic activity is strongly dependent on protein dynamics and the displacement of water molecules to accommodate the substrate, consistent with the decreased activity observed in the perdeuterated variants.

The disorder observed in the structures is evidently linked to the intricate networks of hydrogen bonds. However, only a few regions of the models show distinct disorder due to variations in the hydrogen-bond patterns. These are the cases of the Thr40 N–Lys1 O and Gly22 N–Asn19 O hydrogen bonds and the His15 side chain. The differences observed in the Thr40 N–Lys1 O interaction are due to the presence of Gly0 at the N-terminus of D-HEWL_EC_, leading to increased disorder. In the case of the Gly22 N–Asn19 O hydrogen bond, the alternate conformation of Asn19 is favoured by the side-chain disorder of Gln41 that is present in D-HEWL_PP_ and H-HEWL, resulting in a weaker Gly22 N–Asn19 O inter­action. Finally, the His15 side-chain disorder, with differentiation between the two conformational networks *A* and *B*, appears to be linked to partial occupancies of waters 57 and 81 and of nitrate ion 9, and potentially to variations in protonation states. In D-HEWL_PP_, the absence of water 81 seems to promote the disorder of Thr89 and subsequently the flipping of Asp87 to stabilize His15*A*. The protonation states are not evident, even in the 0.65 Å resolution structure (Wang *et al.*, 2007 ▸), and obtaining an unambiguous picture of the proton­ation of lysozyme will require high-quality and high-resolution neutron diffraction data. In conclusion, these minor variations in the protein structure alone are not likely to explain the decrease in stability observed in the D-HEWL structures.

The main difference in the crystal structures that can be correlated with variations in protein stability is the disorder of the Lys97–Gly104 region due to the partial peptide-plane flip of Asn103. Peptide-plane flipping occurs in the early stages of protein folding, particularly when glycine is in the *i* + 1 position, since the structure is not yet restrained by hydrogen bonds between protein residues (Hayward, 2001 ▸). Although not frequent due to its energetically unfavored conformation, peptide flipping remains underrepresented in the PDB (Berman *et al.*, 2000 ▸). This was found to be correlated, among other factors, with the resolution of the X-ray data available to determine the crystal structures (Stewart *et al.*, 1990 ▸; Weiss *et al.*, 1998 ▸). Peptide flipping can be responsible for amyloid formation (Milner-White *et al.*, 2006 ▸; Yang *et al.*, 2006 ▸) or can confer structural flexibility that is essential for protein function (Weiss *et al.*, 1998 ▸; Ludwig *et al.*, 1997 ▸; Keedy *et al.*, 2015 ▸). As described by Wang *et al.* (2007 ▸), the backbone disorder in this region is a consequence of the Asn103 peptide-plane flip. In their H-HEWL crystal structure determined from X-ray data at 0.65 Å resolution, the flipped conformation has a refined occupancy of 35%, which is consistent with our H-HEWL model in which the flipped conformation of Asn103 was refined with an occupancy of 33%. This observation suggests that the likelihood of Asn103 peptide flipping in native H-HEWL is constant. Conversely, in the D-HEWL models the refined occupancies for the flipped conformation are greater: 46% in D-HEWL_EC_ and 38% in D-HEWL_PP_. While D-HEWL_EC_ was chemically unfolded and then refolded by slowly changing its buffer from 6 *M* guanidine–HCl to a 2 *M* urea H_2_O solution, D-HEWL_PP_ was folded in deuterated conditions during expression. Thus, both D-HEWL variants were subjected to different folding environments compared with H-HEWL, which are associated with slower solvent dynamics and the H/D-isotope effect, which could favour the peptide-plane flip of Asn103. Interestingly, when the protein is completely unfolded, as is the case for D-HEWL_EC_, it appears that the probability of the peptide flip occurring or not is identical, suggesting a high degree of freedom between the two conformations. In the case of D-HEWL_PP_, the solvent-isotope effect may be responsible for this by slowing down the folding dynamics and increasing the likelihood of peptide flipping. This destabilized region is not only part of the enzyme active site, and therefore relevant to substrate binding, as reported by Strynadka & James (1991 ▸), but also protects a hydrophobic pocket containing Trp28, Trp62, Trp63 and Trp108. The increase in disorder of this loop region may therefore be correlated with the decrease in protein thermal stability measured for D-HEWL_EC_ when compared with D-HEWL_PP_.

The results presented here support the widespread understanding that perdeuteration has no significant effect on secondary and tertiary protein structures. Nevertheless, the hydrophobic effect and the slower dynamics caused by perdeuteration have an impact on protein stability and activity. Ultimately, this study emphasizes the capability to use *E. coli* for the expression of recombinant insoluble protein and subsequent refolding for the production of large amounts of perdeuterated material, enabling a wide range of new science in the future. In addition, this work highlights the fact that studies of deuterated proteins can reveal crucial and highly specific aspects of protein conformation related to variations in protein thermal stability.

## 5. Data accessibility   

The X-ray diffraction data and models have been deposited in the PDB with accession codes 7ave (D-HEWL_EC_), 7avf (H-HEWL) and 7avg (D-HEWL_PP_).

## Supplementary Material

PDB reference: hen egg-white lysozyme, hydrogenated, 7avf

PDB reference: perdeuterated, expressed in *E. coli* and refolded, 7ave

PDB reference: perdeuterated, produced in *Pichia pastoris*, 7avg

Supplementary Figures. DOI: 10.1107/S2052252521001299/jt5055sup1.pdf

## Figures and Tables

**Table 1:** X-ray diffraction data-collection and model-refinement statistics for H-HEWL, D-HEWL_EC_ and D-HEWL_PP_ Values in parentheses are for the outer resolution shell.

	H-HEWL	D-HEWL_EC_	D-HEWL_PP_
Cryoprotectant	25%(*v*/*v*) glycerol	35%(*v*/*v*) d_8_-glycerol	30%(*v*/*v*) d_8_-glycerol
Strategy	2 κ orientations, 180° scans	2 κ orientations, 180° scans	2 κ orientations, 360° scans
Beamline and source	I03, DLS	I03, DLS	BioMAX, MAX IV
Detector	EIGER2 XE 16M	EIGER2 XE 16M	EIGER hybrid-pixel 16M
Wavelength (Å)	0.7293	0.7293	0.7999
Resolution range (Å)	31.99–1.00 (1.036–1.000)	32.01–0.98 (1.015–0.980)	32.01–1.00 (1.036–1.000)
Space group	*P*1	*P*1	*P*1
*a*, *b*, *c* (Å)	26.76, 31.07, 33.77	26.67, 30.97, 33.74	26.67, 30.97, 33.74
α, β, γ (°)	89.211, 72.459, 67.863	89.439, 72.818, 67.503	89.439, 72.818, 67.503
Total reflections	178195 (17670)	278571 (24674)	337122 (32144)
Unique reflections	50297 (4908)	52966 (5018)	49991 (4880)
Multiplicity	3.5 (3.6)	5.3 (4.9)	6.7 (6.6)
Completeness (%)	97.47 (94.95)	97.44 (92.67)	97.71 (95.50)
Mean *I*/σ(*I*)	9.25 (2.81)	18.09 (4.21)	15.07 (6.92)
Wilson *B* factor (Å^2^)	8.43	7.12	8.81
*R*_merge_	0.0727 (0.386)	0.0424 (0.273)	0.0803 (0.262)
*R*_meas_	0.0856 (0.453)	0.0470 (0.306)	0.0876 (0.284)
*R*_p.i.m._	0.0449 (0.236)	0.0201 (0.136)	0.0344 (0.109)
CC_1/2_	0.996 (0.884)	0.999 (0.935)	0.992 (0.979)
CC*	0.999 (0.969)	1.00 (0.983)	0.998 (0.995)
Reflections used in refinement	50286 (4906)	52964 (5018)	49982 (4878)
Reflections used for *R*_free_	2398 (218)	2510 (223)	2400 (222)
*R*_work_	0.1172 (0.1577)	0.1049 (0.1323)	0.1205 (0.1206)
*R*_free_	0.1319 (0.1740)	0.1145 (0.1423)	0.1341 (0.1326)
CC_work_	0.976 (0.956)	0.977 (0.969)	0.965 (0.977)
CC_free_	0.976 (0.945)	0.972 (0.963)	0.941 (0.970)
No. of non-H/D atoms			
Total	1502	1467	1474
Macromolecule	1308	1291	1299
Ligands	40	40	40
Solvent	154	136	135
Protein residues	129	130	129
R.m.s.d., bond lengths (Å)	0.011	0.008	0.013
R.m.s.d., angles (°)	1.46	1.42	1.62
Ramachandran favoured (%)	96.85	97.66	97.64
Ramachandran allowed (%)	3.15	2.34	2.36
Ramachandran outliers (%)	0	0	0
Rotamer outliers (%)	0.7	1.44	0.71
Clashscore	4.18	2.32	4.6
Average *B* factor (Å^2^)			
Overall	11.06	9.96	11.84
Macromolecule	10.27	9.19	11.33
Ligands	16.50	16.71	16.55
Solvent	16.37	15.25	15.35

**Table 2:** Expected and observed masses for D-HEWL_EC_ and D-HEWL_PP_ in the MS experiments

Sample	MW of hydrogenated oxidized form (Da)	No. of non-exchangeable H positions	No. of exchangeable H positions	Expected mass of perdeuterated variant in H_2_O (Da)	Observed mass in H_2_O (Da)
D-HEWL_EC_	14362	698	256	15064	15060
D-HEWL_PP_	14305	696	255	15005	15005
