# Supplementary material for: Structural insights into protein folding, stability and activity using in vivo perdeuteration of hen egg-white lysozyme
Source: IUCrJ. 2021 Mar 6;8(Pt 3):372–86. doi: 10.1107/S2052252521001299 (PMC8086161; doi:10.1107/S2052252521001299)
Supplement: Supplementary file 1 [file m-08-00372-sup1.pdf]

IUCrJ

Volume 8 (2021)

Supporting information for article:

Structural insights into protein folding, stability and activity using *in vivo* perdeuteration of hen egg-white lysozyme

Joao Ramos, Valerie Laux, Michael Haertlein, Elisabetta Erba Boeri, Katherine E. McAuley, V. Trevor Forsyth, Estelle Mossou, Sine Larsen and Annette E. Langkilde

a

atg ggt aag gtt ttt ggt cgt tgc gaa ctg gcg gcg gcg atg aag cgt cac ggt ctg gac
(M) G K V F G R C E L A A A M K R H G L D

aat tat cgt ggt tat agc ctg ggt aac tgg gtg tgc gcg gcg aag ttc gag agc aac ttt

N Y R G Y S L G N W V C A A K F E S N F

aac acc cag gcg acc aac cgt aac acc gac ggt agc acc gat tac ggc atc ctg caa att

N T Q A T N R N T D G S T D Y G I L Q I

aac agc cgt tgg tgg tgc aac gat ggt cgt acc ccg ggc agc cgt aac ctg tgc aac atc

N S R W W C N D G R T P G S R N L C N I

ccg tgc agc gcg ctg ctg agc agc gac att acc gcg agc gtg aac tgc gcg aag aaa atc

P C S A L L S S D I T A S V N C A K K I

gtt agc gat ggt aac ggc atg aac gcg tgg gtt gcg tgg cgt aac cgt tgc aaa ggc acc

V S D G N G M N A W V A W R N R C K G T

gat gtt cag gcg tgg att cgt ggc tgc cgt ctg taa

D V Q A W I R G C R L -

b

aaa gtg ttt ggc cgt tgc gaa ctg gcg gcg gcg atg aaa cgt cat ggc ctg gat aac acc

K V F G R C E L A A A M K R H G L D N T

cag gcg acc aac cgt aac acc gat ggc agc acc gat tat ggc att ctg cag att aac tat

Q A T N R N T D G S T D Y G I L Q I N Y

cgt ggc tat agc ctg ggc aac tgg gtg tgt gcc gcc aaa ttt gaa agc aac ttc aac agc

R G Y S L G N W V C A A K F E S N F N S

cgt tgg tgg tgt aac gat ggc cgt acc ccg ggc agc cgt aac ctg tgt aac att ccg tgt

R W W C N D G R T P G S R N L C N I P C

agc gcc ctg ctg tct agc gat att acc gcc agc gtg aac tgt gcc aaa aaa att gtg agc

S A L L S S D I T A S V N C A K K I V S

gat ggc aac ggc atg aac gcc tgg gtg gcg tgg cgt aac cgt tgt aaa ggc acc gat gtt

D G N G M N A W V A W R N R C K G T D V

cag gcc tgg att cgt ggc tgc cgt ctg taa

Q A W I R G C R L -

Figure S1

DNA coding and protein sequences for the recombinantly expressed proteins, D-HEWL$_{EC}$ (a) and D-HEWL$_{PP}$ (b).

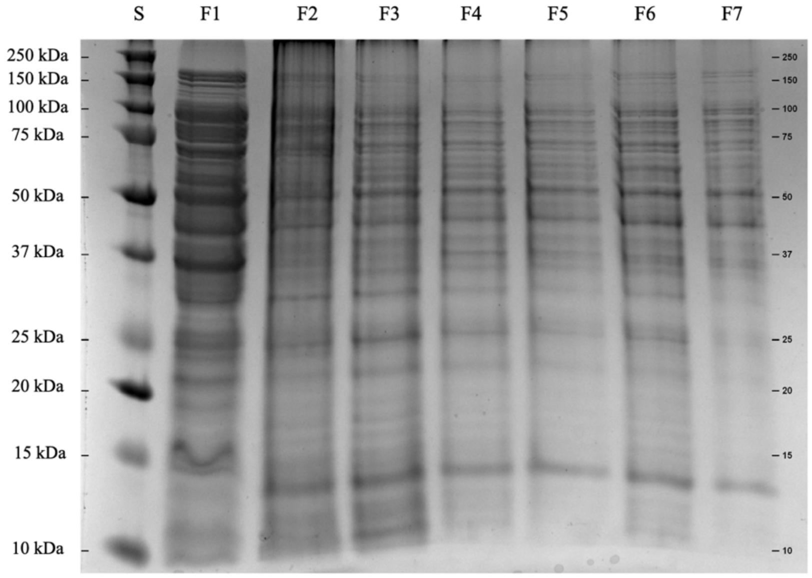

Figure S2

Samples from the inclusion body washing steps in a 12% SDS-PAGE gel – lanes: S. Precision Plus Protein™ Dual Xtra Standards (Bio-Rad); F1. Soluble fraction from cell lysis; F2. Soluble fraction from 1st washing cycle; F3. Soluble fraction from 2nd washing cycle; F4. Soluble fraction from 3rd washing cycle; F5. Soluble fraction from 4th washing cycle; F6. Soluble fraction from 5th washing cycle; F7. Soluble fraction from 6th washing cycle.

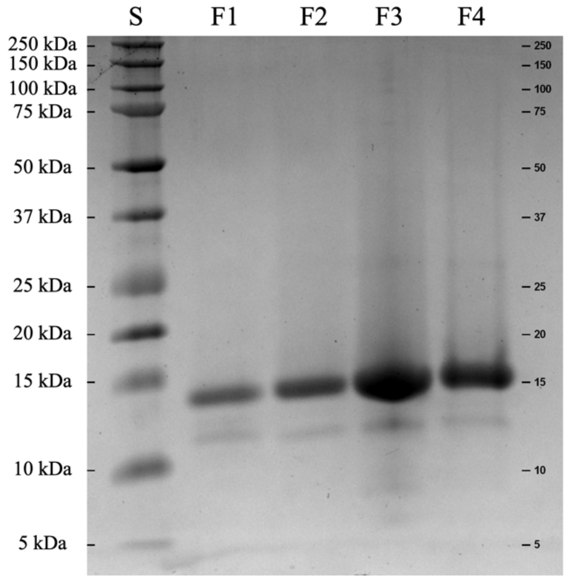

Figure S3

Samples from D-HEWL refolding SEC and desalting to deuterated protein buffer in a 12% SDS-PAGE gel – lanes S. Precision Plus Protein™ Dual Xtra Standards (Bio-Rad); F1. Fractions from refolding SEC before 0.9 CV; F2. Refolded monomeric D-HEWL in refolding buffer; F3. Refolded monomeric D-HEWL in protein buffer in D_2O; F4. Diluted sample of F3.

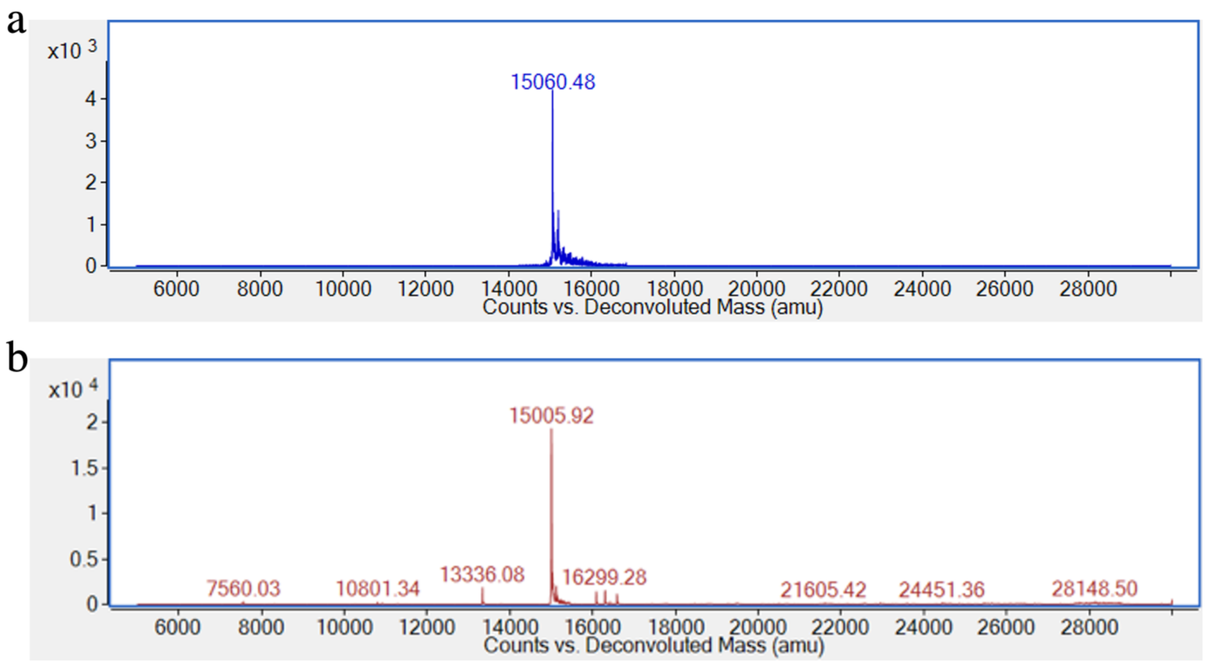

Figure S4

Mass spectra of D-HEWL$_{EC}$ (a) and D-HEWL$_{PP}$ (b) from LC/ESI-MS analysis. The most abundant masses observed were of 15060 and 15005 Da, for D-HEWL$_{EC}$ and D-HEWL$_{PP}$, respectively.

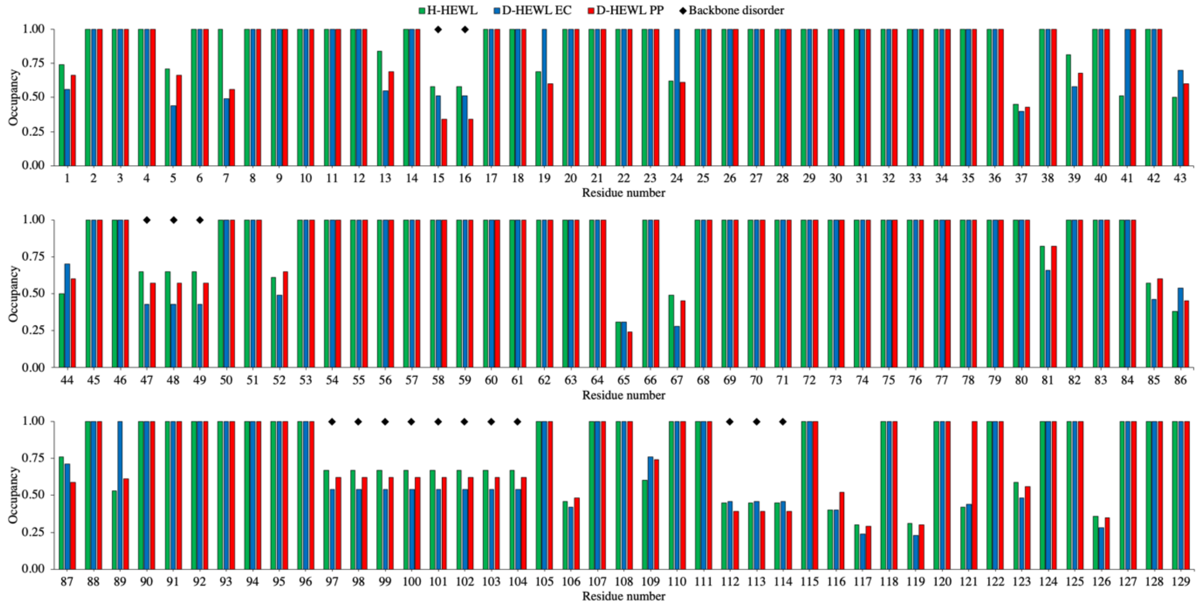

Figure S5

Residue occupancies of conformation A by residue number in H-HEWL (green), D-HEWL$_{EC}$ (blue) and D-HEWL$_{PP}$ (red). An occupancy of 1 represents single confirmations, while occupancies <1 indicate alternate residue conformations (modelled with a total residue occupancy of 1). Regions containing backbone disorder are highlighted (♦).

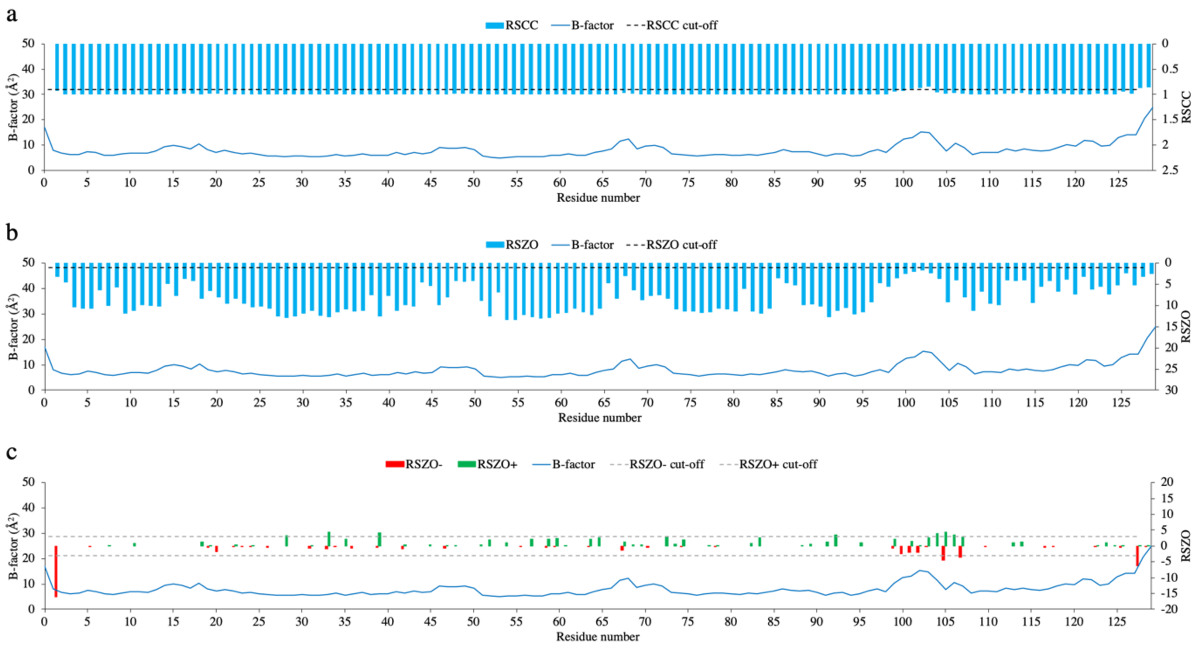

Figure S6

Validation of the quality of the D-HEWL$_{EC}$ model against the X-ray data, according to the RSCC (a), RSZD (b), RSZD- and RSZD+ (c), metrics from EDSTATS. The cut-offs applied were: 90% for RSCC, 1 σ for RSZD, -3 σ for RSZD- and +3 σ for RSZD+. In the D-HEWL$_{EC}$ model, the residues excluded from the detailed H-bond analysis were Gly0, Asn27, Ala32, Phe38, Val92, Asp101, Gly102, Asn103, Gly104, Met105, Asn106, Ala107, Arg128 and Leu129.

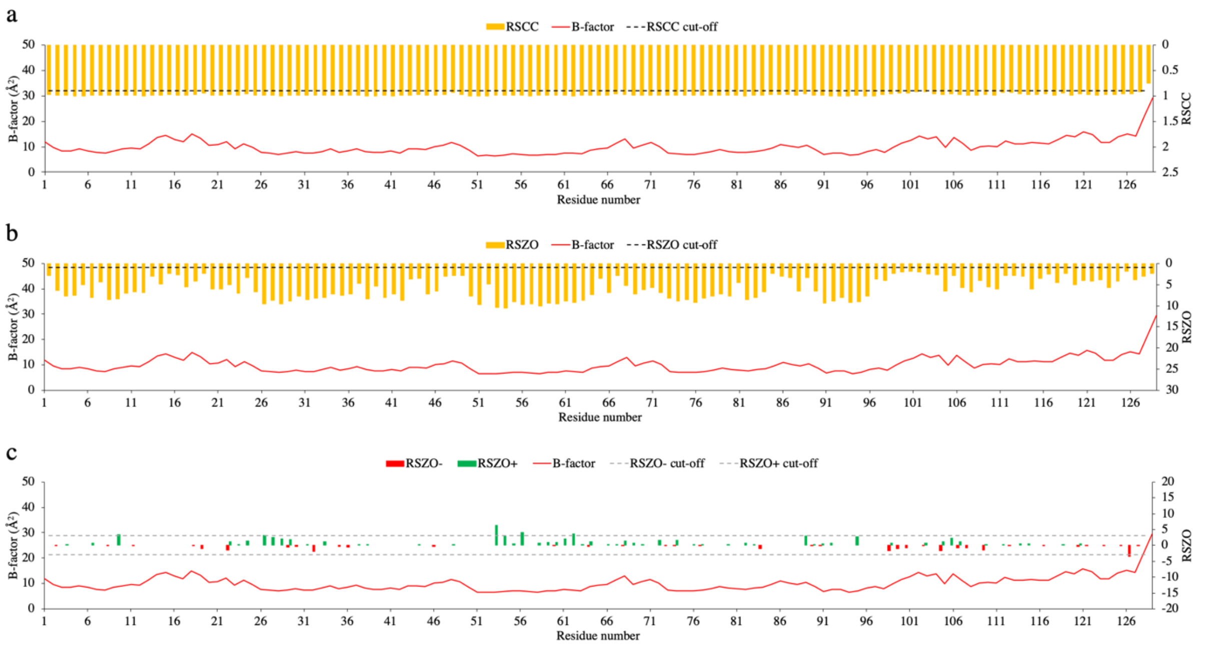

Figure S7

Validation of the quality of the D-HEWL$_{PP}$ model against the X-ray data, according to the RSCC (a), RSZD (b), RSZD- and RSZD+ (c), metrics from EDSTATS. The cut-offs applied were: 90% for RSCC, 1 σ for RSZD, -3 σ for RSZD- and +3 σ for RSZD+. In the D-HEWL$_{PP}$ model, the residues excluded from the detailed. H-bond analysis were Ala9, Gly26, Tyr53, Leu56, Trp62, Thr89, Cys127 and Leu129.

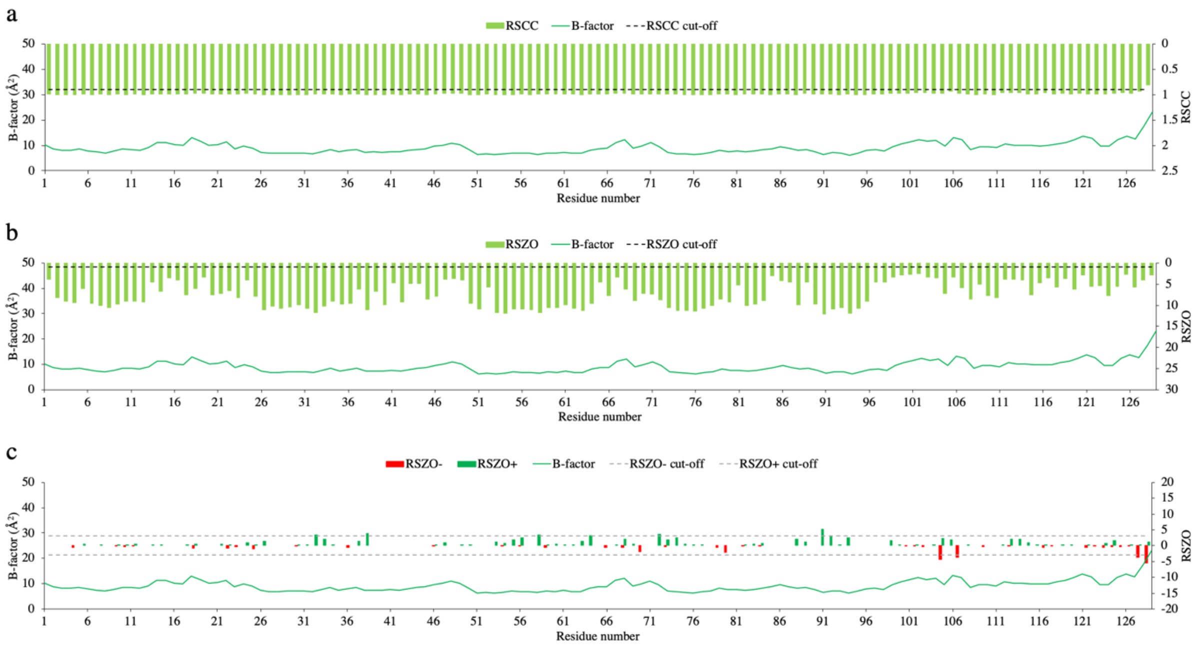

Figure S8

Validation of the quality of the H-HEWL model against the X-ray data, according to the RSCC (a), RSZD (b), RSZD- and RSZD+ (c), metrics from EDSTATS. The cut-offs applied were: 90% for RSCC, 1 σ for RSZD, -3 σ for RSZD- and +3 σ for RSZD+. In the H-HEWL model, the residues excluded from the detailed H-bond analysis were Ala32, Phe38, Ile58, Cys64, Ser72, Ser91, Met105, Ala107, Arg128 and Leu129.

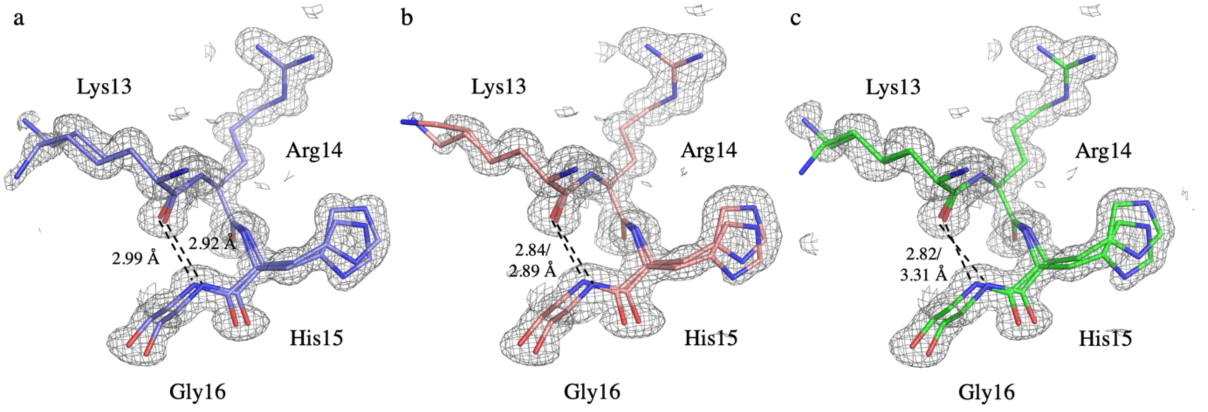

Figure S9

Representation of the Lys13-Gly16 backbone disorder and variation in the Gly16(N)-Lys13(O) H-bond between D-HEWL$_{EC}$ (a), D-HEWL$_{PP}$ (b) and H-HEWL (c). The 2F$_o$-F$_c$ electron density map represented is contoured at 1 σ.

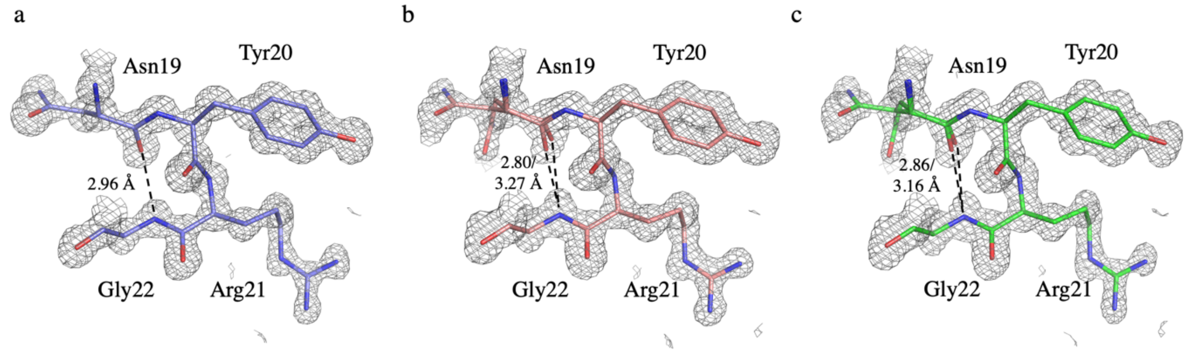

Figure S10

Representation of the differences in Gly229(N)-Asn19(O) H-bond between D-HEWL$_{EC}$ (a), D-HEWL$_{PP}$ (b) and H-HEWL (c). The 2F$_o$-F$_c$ electron density map represented is contoured at 1 σ.

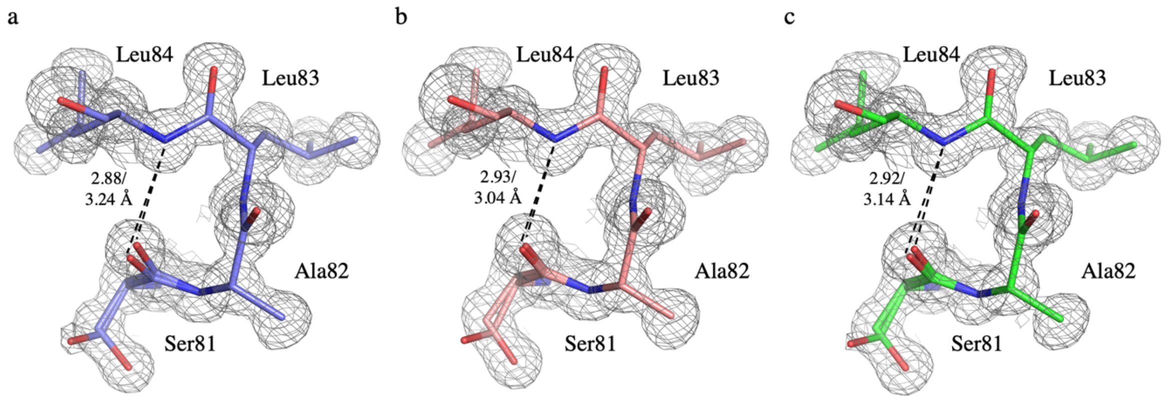

Figure S11

Representation of the differences in the Ser81(N)-Leu84(O) H-bond between D-HEWL$_{EC}$ (a), D-HEWL$_{PP}$ (b) and H-HEWL (c). The $2F_o$-F_c electron density map represented is contoured at 1 σ.

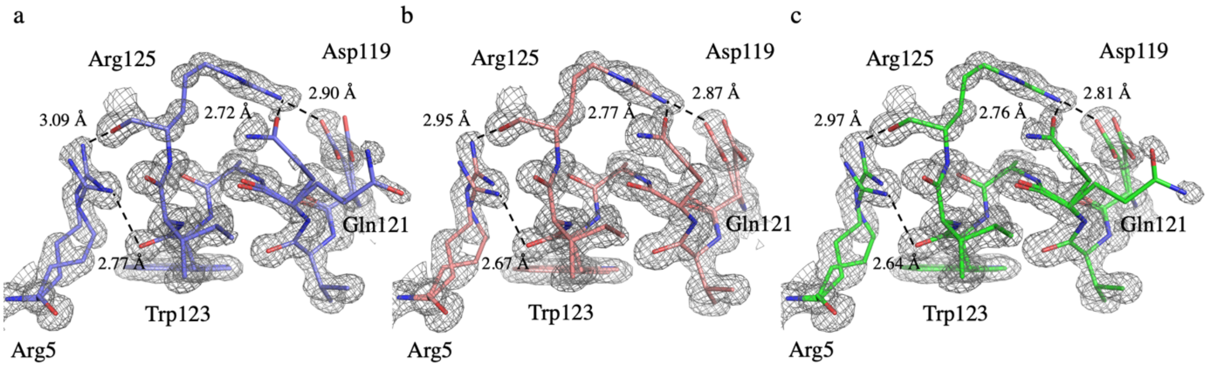

Figure S12

Representation of the differences in the H-bond pattern, involving Arg5(NH1 and NH2), Trp123(O), Arg125(O and NH2), Asp 119(OD2) and Gln121(OE1), between D-HEWL$_{EC}$ (a), D-HEWL$_{PP}$ (b) and H-HEWL (c). The $2F_o$-F_c electron density map represented is contoured at 1 σ.
